# Non-communicable diseases in the southwest of Iran: profile and baseline data from the Shahrekord PERSIAN Cohort Study

**DOI:** 10.1186/s12889-021-12326-y

**Published:** 2021-12-13

**Authors:** Ali Ahmadi, Majid Shirani, Arsalan Khaledifar, Morteza Hashemzadeh, Kamal Solati, Soleiman Kheiri, Mehraban Sadeghi, Abdollah Mohammadian-Hafshejani, Hadi Raeisi Shahraki, Alireza Asgharzadeh, Ali Zamen Salehifard, Masoumeh Mousavi, Elaheh Zarean, Reza Goujani, Seyed Saeed Hashemi Nazari, Hossein Poustchi, Pierre-Antoine Dugué

**Affiliations:** 1 Modeling in Health Research Center, Shahrekord University of Medical Sciences, P.O. Box: 881-55383, Shahrekord, Iran; 2 Department of Epidemiology and Biostatistics, School of Public Health, Shahrekord University of Medical Sciences, P.O. Box: 881-55383, Shahrekord, Iran; 3 Department of Urology, Shahrekord University of Medical Sciences, Shahrekord, Iran; 4 Department of Cardiology, School of Medicine, Shahrekord University of Medical Sciences, Shahrekord, Iran; 5 Cellular and Molecular Research Center, Shahrekord University of Medical Sciences, Shahrekord, Iran; 6 Department of Psychiatry, Shahrekord University of Medical Sciences, Shahrekord, Iran; 7 Department of Environmental Engineering, School of Public Health, Shahrekord University of Medical Sciences, Shahrekord, Iran; 8 School of Medicine, Shahrekord University of Medical Sciences, Shahrekord, Iran; 9 Safety Promotion and Injury Prevention Research Center, Department of Epidemiology, School of Public Health and Safety, Shahid Beheshti University of Medical Sciences, Tehran, Iran; 10 Liver and Pancreatobiliary Diseases Research Center, Digestive Diseases Research Institute, Tehran University of Medical Sciences, Tehran, Iran; 11 Precision Medicine, School of Clinical Sciences at Monash Health, Monash University, Clayton, VIC Australia; 12 Cancer Epidemiology Division, Cancer Council Victoria, Melbourne, VIC Australia; 13 Centre for Epidemiology and Biostatistics, Melbourne School of Population and Global Health, The University of Melbourne, Parkville, VIC Australia

**Keywords:** Cohort, Non-communicable diseases, Risk factors, Multimorbidity, PERSIAN

## Abstract

**Background:**

Critical inter-provincial differences within Iran in the pattern of non-communicable diseases (NCDs) and difficulties inherent to identifying prevention methods to reduce mortality from NCDs have challenged the implementation of the provincial health system plan. The Shahrekord Cohort Study (SCS) was designed to address these gaps in Chaharmahal and Bakhtiari, a province of high altitude in the southwest of Iran, characterized by its large Bakhtiari population, along with Fars and Turk ethnicity groups.

**Methods:**

This ongoing cohort, a prospective, large-scale longitudinal study, includes a unique, rich biobank and was conducted for the first time in Chaharmahal and Bakhtiari Province in Iran. SCS is a part of the PERSIAN (Prospective Epidemiological Research Studies in IrAN) cohort. The study began in 2015, recruited 10075 participants (52.8% female, 47.2% male) from both urban (n=7034) and rural (*n*=3041) areas, and participants will be annually followed up for at least 15 years. A cross-sectional analysis was conducted using baseline data from the SCS, using descriptive statistics and logistic regression. Data analysis was performed using Stata software.

**Results:**

The prevalence of NCDs was 9.8% for type 2 diabetes, 17.1% for hypertension, 11.6% for thyroid disease, 0.2% for multiple sclerosis and 5.7, 0.9 and 1.3% for ischemic heart disease, stroke and myocardial infarction, respectively. The prevalence of multimorbidity (≥2 NCDs) was higher in women (39.1%) than men (24.9%). The means (standard deviations) of age, BMI, systolic blood pressure and fasting blood glucose were 49.5 (9) years, 27.6 (4.6) kg/m^2^, 115.4 (17.3) mmHg and 96.7 (27.3) mg/dL, respectively. Logistic regression models showed that older age, female gender, living in an urban area, non-native ethnicity, high wealth index, unemployment, obesity, low physical activity, hypertriglyceridemia, high fasting blood sugar, alkaline urine pH and high systolic and diastolic blood pressure were associated with increased prevalence of NCDs.

**Conclusions:**

The SCS provides a platform for epidemiological studies that will be useful to better control NCDs in the southwest of Iran and to foster research collaboration. The SCS will be an essential resource for identifying NCD risk factors in this region and designing relevant public health interventions.

## Background

The World Health Organization (WHO) estimates that more than 36 million people worldwide die annually from non-communicable diseases (NCDs) (63% of all deaths), and 14 million of these deaths occur before the age of 70. More than 90% of premature deaths from NCDs occur in low- and middle-income countries [1]. Reducing the prevalence, incidence, mortality, and the costs and burden associated with NCDs, especially cardiovascular disease and cancer, is, therefore, a significant global challenge and priority for public health [2, 3]. Increased urbanization, industrialization, adoption of modern lifestyles, and rising life expectancy have increased exposure to risk factors and the occurrence of NCDs [4, 5]. Obtaining a better description and understanding of NCD risk factors and trends in Iran is a priority for the government [5] as it will allow achieving the action plan for the prevention and control of NCDs 2013-2020 set by the WHO (resolution WHA66.10) [6].

Iran is a developing country with a population of over 80 million. In the last two decades, the epidemiological features of health and disease in Iran have changed dramatically due to significant variations in demographic indices and health-related social and economic factors [7]. These changes have led Iran's health system to prioritize preventing NCDs over controlling communicable diseases [3, 5]. Currently, NCDs impose the heaviest burden on Iran's health system. In 2020, according to WHO reports, 82% of deaths in Iran were caused by NCDs (43% cardiovascular diseases, 16% cancers, and 23% other NCDs) [8]. Substantial differences exist between Iran and other Western and Eastern Mediterranean countries in terms of ecological, cultural, and social characteristics. There are also critical inter-provincial differences within Iran in the pattern of health and disease [7]. As one of the principal aims of the WHO is to reduce mortality from NCDs worldwide by 25% by 2025, establishing a national and regional plan to control these diseases, and supporting and conducting research in this region, is a significant public health objective. These WHO future goals have, therefore, set the perfect stage for creating high-quality regional documentation to inform decision-making by health system planners and better prevent, control, and manage these fatal diseases [8]. Several isolated cohort studies have been previously conducted in Iran to address NCDs in various regions and ethnicities, such as the Golestan Cohort Study including Turkmen [9], the Amirkolah Health and Ageing Project in northern Iran [10], the Yazd Health Study in central Iran [11], as well as the Tehran Glucose and Lipid Study in the capital city [12]. However, the Prospective Epidemiological Research Studies in IrAN (PERSIAN) is the most extensive, multi-center cohort to fulfill this purpose [13]. The PERSIAN Cohort started in 2014 in 19 centers, intending to include all the major ethnic groups in various regions of Iran, including Kurds [14], Turks [15], Fars [16], Tabari [17], and Arabs, among other ethnicities [13]. No cohort study was previously conducted in the Bakhtiari ethnicity; therefore, the Shahrekord Cohort Study (SCS), as one of the PERSIAN Cohort Centers, has filled this gap to assess health patterns and risk factors in people of Bakhtiari ethnicity [18]. The SCS was therefore conducted to study NCDs in an Iranian province with distinctive environmental, geographical (the highest region above sea level in Iran), and ethnic and social (Bakhtiari) characteristics compared with other Iranian areas and the rest of the world.

The Shahrekord Cohort Study may have consequences for the appropriate interventions to prevent and manage NCDs in this region. Despite recent efforts to investigate NCDs in Iran [4, 9–17], there are currently no comprehensive, population-based and reliable data sources to obtain accurate health information in this province to design better management plans for improving the health care system. The aims of the SCS study are, therefore: i) to evaluate the prevalence and long-term trends of NCDs and their outcomes in an Iranian province with unique geographical, ethnic, and socioeconomic characteristics, ii) to investigate associations of environmental and genetic/ethnic factors with the prevalence and incidence of NCDs and their outcomes, iii) to examine the interplay between genetic/ethnic and environmental factors in the etiology and prevention of NCDs, iv) to provide the basis for various types of epidemiological studies (e.g., social, spatial, molecular epidemiology) and generate scientific evidence that may contribute to improving public health in the Chaharmahal and Bakhtiari (CH&B) province, v) to provide a research and education platform and a resource for national and international collaboration and to make the research community aware of the existence of large cohorts around the world.

## Methods

This population-based prospective cohort study recruited participants from the CH&B province in Southwest Iran. A total of 10075 participants were recruited from the districts of Shahrekord and Ardal, situated in urban (7034 participants) and rural (3041 participants) areas, respectively. We used baseline enrolment data (the first wave of measured exposures and outcomes) from 6 Oct 2016 to 22 Aug 2019 to generate preliminary results. The second wave of the reassessment phase was started in September 2021. That study was approved by the Ethics Committee of the Shahrekord University of Medical Sciences (IR.SKUMS.REC.1400.073)

### Setting

The Bakhtiari ethnic group mainly lives in Chaharmahal and Bakhtiari (CH&B) province in Iran and has an estimated population of 1.25 million. It is a subgroup of the Iranian Lurs, and the genetic background of Bakhtiari people is different from other Lur populations [19]. They speak the Bakhtiari dialect. The Bakhtiari have maintained their bloodlines primarily intact over the centuries, mainly marrying within their tribe. Other notable differences with other Iranian ethnic groups include their culture and social and local customs (mourning and weddings), and dietary habits (tiri bread, mountain vegetables, animal oil consumption, traditional dairy products), type of employment (animal farming, herding, agriculture, hunting), clothing and apparel (local dress), and different environmental exposures, such as exposure to sunlight at high altitudes. CH&B province covers an area of 16.421 km^2^ and is situated in the southwest of Iran, north of the Zagros Mountains, which have the highest average elevation above sea level in Iran; the Shahrekord region is known as the “roof of Iran”. Despite its relatively small area (1% of the total area of Iran), CH&B holds 10% of the country’s water resources. Because of its mountainous nature where moist Mediterranean air converges, this province has relatively abundant rainfall [18, 19]. Because of the rare ethnic groups (Bakhtiari), Fars, and Turk living in this region, this cohort is unique in Iran and worldwide [18].

### Inclusion and exclusion criteria

Participants were required to be aged between 35 and 70 years at the time of recruitment, having lived in the specified area for at least one year, having completed and signed the informed consent, and having Iranian nationality (i.e., having an Iranian birth certificate and a national identification number).

Both gender (male and female) and pregnant women were included. The 35-70 years age range was chosen because people in this age group are more likely to have well-established behaviors and lifestyles, are active enough to participate in a cohort study and have a sufficiently high risk of developing NCDs during the study [13, 18]. People unable to undertake the required questions and measures (e.g., due to disability or mental disorders) were not eligible for the study.

### Subjects and public involvement

The numbers of invited and recruited SCS participants, the area names in these districts, and the region map are presented on the SCS protocol and website. The formula used for calculating the study's sample size was described previously in the SCS protocol [18]. The implementation, feasibility, and sampling processes of the SCS were performed in the pre-pilot phase of the study from 22 Nov 2015 to 10 Sept 2016. In this phase, the data collection process and biological sample storage capacity were evaluated. Aspects of the regulation of ownership, preservation, and storage of data were also finalized. The pre-pilot phase also allowed i) to evaluate the participants' response rate and the recruitment and training of interviewers, ii) to determine the frequency of participant follow-up contacts, iii) to check the validity of measurements, and iv) to implement adequate procedures for bio-sampling and quality control of the collected data. The study protocols were revised accordingly to improve the validity and reliability of the questionnaires and the acceptability of the data collection techniques [13, 18].

The pilot phase was conducted from 6 Oct 2016 to 20 Dec 2016 to further evaluate the study protocol's main strengths and weaknesses and participant recruitment process. A total of 1000 participants aged 35–70 years were recruited for this phase. After receiving confirmation from the quality control team, the main stage, enrolling all participants, started with the pilot phase on 6 Oct 2016. Checklists about several study processes (invitation process and evaluation of response rate, questionnaires, sampling and measurements, assessment of ascertainment methods, establishment of follow-up data processes, testing the validity of disease outcomes) were completed. The quality assurance team issued a certificate of study commencement after the infrastructure and procedures were in place.

The multistage sampling method (stratified proportional cluster sampling) was applied to recruit participants in the SCS in the pilot and main phase. According to the national census statistics, 70% of the CH&B province population lives in urban areas, and 30% live in rural areas, so the urban and rural strata accounted for 70% and 30% of the study sample size respectively [18]. Sampling in urban areas was also carried out using the cluster sampling method. Each of the four regions of Shahrekord County (the capital city of CH&B) was considered an eligible cluster. Then a specific geographical region of Shahrekord was randomly chosen as the cohort cluster. The population covered by each urban healthcare center (cluster) was used as sampling weights for recruiting participants from the corresponding (*n*=77030) health care centers to constitute the final study sample for the urban area (*n*= 7034). For rural areas, the sampling process was as follows: i) nine CH&B counties were considered as eligible clusters, ii) the Ardal county was randomly selected as the cohort cluster, and iii) three of the five rural clusters of the Ardal county were randomly selected for inclusion in the SCS. A total of 3041 participants from these rural areas were recruited based on census-collected information on healthcare coverage provided by the health centers and health houses. The first contact with prospective SCS participants was made through an invitation by phone to eligible people, and this process continued until the required sample size was met. Several initiatives were taken to increase participant enrolment and satisfaction, such as inviting people to visit the center, friendly staff and interviewers, and the provision of a suitable space for participants to undertake data collection. All medical examinations and tests were free of charge, and the results of the tests were provided to the participants. Less than one percent of the people contacted declined to participate in the study, and only 20 participants dropped out. Living areas and sex- and age-specific proportions of the participants included in the SCS were cross-checked against and found by the national population figures provided by the national census conducted by the Statistical Center of Iran. The distribution of the main socio-demographic characteristics of SCS participants is shown in Table 2.

### Measurements

Data collection in SCS, including the definition of NCDs, used the PERSIAN cohort protocols [13, 18]. In brief, the following NCDs were considered: heart diseases (myocardial infarction, ischemic heart disease), hypertension, endocrine conditions (diabetes mellitus, hypo/hyperthyroidism), neurological diseases (stroke/transient ischemic attack, epilepsy, Parkinson’s disease, migraine, chronic headache, Alzheimer’s disease/dementia, multiple sclerosis), chronic kidney disease, musculoskeletal diseases (arthritis, osteoporosis), respiratory diseases (chronic obstructive pulmonary disease, asthma), gastrointestinal conditions (peptic ulcer, Chron’s disease, ulcerative colitis, fatty liver diseases), cancers, mental and psychological disorders (depression, anxiety). Diabetes was diagnosed as fasting blood sugar (FBS) ≥ 6.99 mmol/L (126 mg/dL) or receipt of blood glucose-lowering treatment. In addition, individuals who were under treatment for physician-diagnosed diabetes were considered to have diabetes. Hypertension was defined as systolic blood pressure ≥140 mmHg or diastolic blood pressure ≥90 mmHg or a prior diagnosis of hypertension or being on antihypertensive medication. Other chronic diseases were self-reported (participant’s response to the question; ‘Has a doctor ever told you that you have any of health problems or presence/absence of NCDs?’), medical documents, clinical history, interview and physician diagnosis [13, 18, 20]. Anthropometric variables including height (in centimeters), weight (in kilograms), waist and hip circumferences (in centimeters) were measured using US National Institutes of Health protocols [13, 18].

The height of participants was measured using a Seca 206 stadiometer and their weight was measured using a Seca analog scale. A standard tape meter was used to measure the participants’ wrist, hip, and waist circumference. Body mass index (BMI, kg/m^2^) was calculated. Physical activity information was collected using the general questionnaire, and self-reported daily activities were converted to metabolic equivalent rates (METs). Blood pressure and pulse rate measures were obtained using a standard barometer (Richter Japan). Tobacco smoking (including tobacco type), drug use (including type of illicit drug), and alcohol use in the last thirty days and one year, respectively. Alcohol use was assessed as standard drinks (one standard drink =100ml wine, 285ml beer or 30ml spirit/toddy/arrack). A consumption of at least six and four standard drinks for men and women, respectively, on at least one occasion during the last 30 days, was considered harmful alcohol use. Based on the laboratory kits used (Pars Azmoun), hypercholesterolemia was defined as having a total cholesterol level of ≥240mg/dl or currently using lipid-lowering drugs. Hypertriglyceridemia was defined as having an overall triglyceride level of ≥150mg/dl or currently using lipid-lowering drugs. According to the American Association for Clinical Chemistry, the average value for urine pH is 6.0, but it can range from 4.5 to 8.0. Urine pH under 5.0 is acidic, and urine pH higher than 8.0 is alkaline or basic. Socio-economic status was defined based on education and wealth index. Education was defined in 5 levels: no schooling (<1 year of primary school), primary school (1-5 years), middle school (6-8 years), high school (9-12 years), and university (>12 years). The wealth index was calculated using multiple correspondence analysis (MCA) on household assets and divided into five quintiles [13, 18, 21]. The tools were used for baseline data collection from 2015 to 2019 and are described in Table 1. In addition to the extensive standard PERSIAN questionnaires, additional questionnaires unique to SCS were also completed, including General Health [22], WHO Quality of Life-BREF [23], Chronic Stressors and Coping Strategies [24], WHO MONICA and the ROSE Angina questionnaire [25], Social Capital [26], Screening Tool for Joint Pain and Musculoskeletal Diseases [27], Health Literacy [28], Oxford Happiness [29], and Oswestry Low Back Pain Disability [30], following SCS protocols [18] in CH&B (Table 1) and additional data unique to SCS were also completed including body composition variables included total body water, body fat mass and percentage, and muscle thickness, which were measured using a body composition analyzer (Tanita, Japan). A spirometry test (pulmonary function test) was performed using the Spirometer device (Spirolab [MIR, Italy)]. An electrocardiography test (ECG) was carried out using an electrocardiogram device (Cardiax®, USA). Although the validity of these questionnaires was assessed in previous studies [13–15, 18, 23, 24], SCS's experts further assessed their face validity and approved their use in the pre-pilot phase of the study.

**Table 1:** Questionnaires and research tools used in the Shahrekord PERSIAN Cohort Study

Dimension	Items	Assessment	Sample	Components/ exposures
General [13, 18]	157	Whole cohort	10075	Age, sex, education, employment status and history, spouse's employment, marital status and number and type of marriages (first-degree or second-degree familial marriage or none); anthropometric measures: weight, height, waist & hip circumference, residence area, domestic and international travels, use of landline and mobile phones, internet access, smoking history; exposure to passive smoking, alcohol consumption, physical activity, sleep duration and quality, daily activities, occupational exposures, cooking and heating fuel, dwelling status, living arrangements, hygiene status of the dwelling and its facilities, drinking water source, history of exposure to animals, agricultural toxins and household pesticides, annual reading rate, access to a freezer, access to a washing machine, access to a dishwasher, access to a computer, access to the internet, access to a motorcycle, access to a car (no access, access to a car with price of <50 million tomans, and access to a car with price of >50 million tomans), access to a vacuum cleaner, color television (TV) type (no color TV or regular color TV vs. Plasma color TV), owning a mobile, owning a PC or laptop, international trips in a lifetime (never, pilgrimage, both pilgrimage and non-pilgrimage trips)
Nutrition [13]	153	Whole cohort	10075	Food Frequency Questionnaire (FFQ), including 153 items; dietary habits during the past year and current; food preparation and storage techniques
Medical history and examination [13]	185	Whole cohort	10075	Current and past medical history, family history of diseases, self-rated health, history of falls and fractures, pain, digestive symptoms, angina, neurological symptoms, history of transfusion, oral health condition, current use of medications, use of drugs, pulse rate and blood pressure measurement plus complete physical examination
General health (GHQ12) [22]	12	Subgroup	7585	Psychological distress, social dysfunction, ability to concentrate, sleep deprivation, ability to make decisions, feeling under stress, ability to overcome difficulties, enjoying healthy activities, facing up problems, feeling unhappy and depressed, losing confidence, thinking of self as worthless, feeling reasonably happy
Quality of life (WHO-QOL) [23]	21	Subgroup	7924	Physical health, mental health, social health, environmental health, self-esteem, interpersonal relationships, sexual activity, social support, home environment, health care, transport, pain, work capacity, mobility, daily activities, leisure activities, financial support, bodily image, security, access to information
Chronic stressor and coping strategies [24]	46	Subgroup	6890	Stress domains consist of household stress, financial pressure, social relationships, personal and professional conflicts, educational concerns, job security, loss and separation, sexual life, daily life, health concerns, exercising, seeking religious support, focusing on the positive, social distancing, acting out, binge-drinking and binge-eating
Modified WHO MONICA [25]	50	Subgroup	7184	Risk factors and treatment history for coronary heart disease
Social capital [26]	44	Subgroup	3080	Memberships, trust, coherence, ability, the value of life, tolerance of diversity, connections, family and friends, neighbors, work colleagues, community participation, feelings of trust and safety, proactivity
Community-oriented program for control of rheumatic diseases [27]	100	Subgroup	3780	Work history, pain/tenderness/swelling/stiffness during the last week, functional disability, difficulty in performing specific tasks, treatment and evaluation, pain scale evaluation, history of NSAID/steroid/ DMARD use, disability
Health literacy [28]	33	Subgroup	5180	Access to and understanding of health information sources
Happiness [29]	29	Subgroup	1904	Feeling healthy, feeling attractive, waking up rested, making decisions quickly, mental alertness, organizational skills, pleased with self, the cheerful effect on others, happy memories, satisfied with life, feeling happy/joyful, feeling committed and involved
Oswestry low back pain [30]	10	Subgroup	4090	Physical function, role-physical and bodily pain indices, vitality, social function

In the pre-pilot phase of the SCS, 100 participants completed the questionnaires. The questionnaire items had coefficients of Cronbach's alpha ranging from 82% to 91%, so they were considered reliable. A complete description of the data dictionary can be found in the http://persiancohort.com/wp-content/uploads/2020/09/PERSIAN-Cohort-Data-Dictionary.pdf.

#### Biological samples and laboratory testing

Sample collection in SCS also followed PERSIAN Cohort protocols [13, 18]. A 25ml blood sample containing whole blood, plasma, and serum was taken at baseline from each participant. Hair samples (around 500 strands, 1 to 3 cm long), nail samples (equal to the number of fingers and toes), and 15 to 25ml urine samples were also taken from participants. Initial hematology tests, biochemical tests, and urine analysis were performed in the SCS laboratory, and the results were provided to the participants for awareness about their health status. All biological samples were then stored at 80°C in the SCS biobank for future use.

### Quality control and assurance

All phases of the study and data collection were monitored by a quality control team, including clinicians, a laboratory specialist, two statisticians, and an epidemiologist, under the supervision of the principal investigators.

### Routine (annual) follow-up

**Fig. 1:**
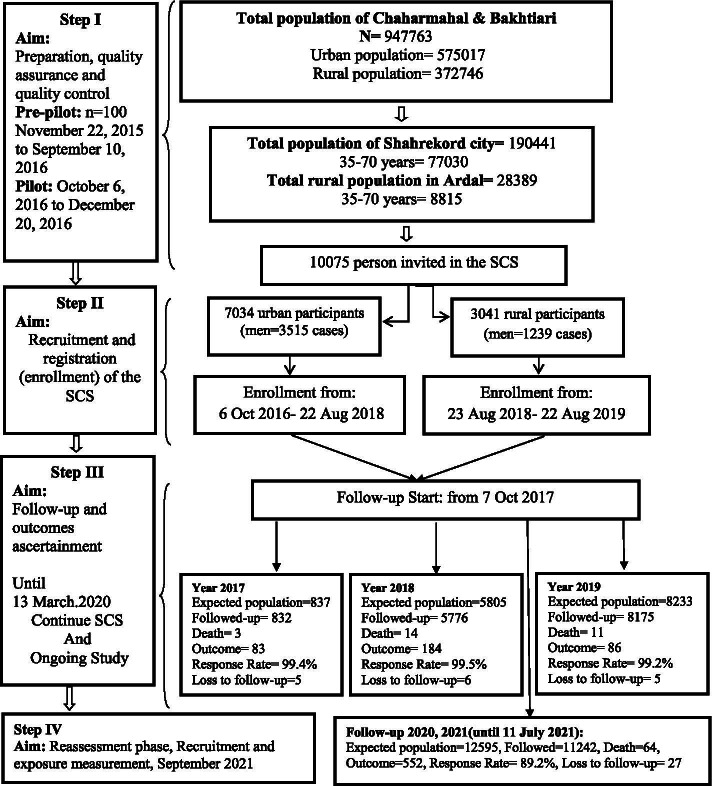
Flow diagram of the phases and data collection timeline of the Shahrekord PERSIAN Cohort Study

The follow-up process aimed to register new cases of common NCDs and their outcomes, including death, cause of death, hospital admissions, and update information on exposures. The SCS focuses primarily on the most common NCDs, including cardiovascular diseases, cancers, and the main endocrine, digestive, hepatic, renal, psychiatric, and respiratory disorders, defined using the International Classification of Diseases 10th version (ICD-10). The annual follow-up of the SCS began in October 2017 and included questionnaires, medical examinations, and linkage with other databases (death, cancer registry). The study follow-up is done annually through telephone calls and links with health databases to identify disease outcomes. More specifically, the follow-up of participants is performed in two forms: an active form including phone interviews and face-to-face interviews (when outcomes occur), and an inactive form, including self-reports. Identification of outcomes is made through automatic notifications received from the healthcare system and linkage with other health databases such as the National Disease and Health Outcome Registry Systems [13, 18]. The follow-up is carried out by a team of trained staff under the supervision of an experienced epidemiologist. Three internal medicine physicians and a professional epidemiologist do outcome assessments, including the cause of death identification. During phone call follow-ups, if an interviewer cannot gather the requested information, additional phone calls are made during the three consecutive weeks (up to 5 to 6 rings). Further attempts to collect incomplete data are made during home visits and face-to-face interviews. In rural areas, the data collection process is conducted in local health care units (Health Houses) by health care staff and an experienced epidemiologist. Participants who experience an outcome are invited to undergo an in-person examination. Additional information about the participants' health history is obtained from the Hospital Information System (HIS) and the integrated electronic health system. When a death occurs, the SCS team visits the participant's home and completes an autopsy form. A schematic representation of the phases of the study and the data collection and follow-up process is shown in Figure 1.

### Statistical analysis

All the analyses were performed using data from the baseline measurements of the cohort. All continuous variables were expressed as mean **±** standard deviation (sd) and categorical variables as frequency and percentage. The analyses were stratified by sex and residence type (urban/rural). Two independent sample t-tests and a chi-square test were used to analyze the data. The prevalence of NCDs risk factors was calculated.

‘Multimorbidity’ was defined as the co-existence of two or more chronic diseases in the cohort, and we further categorized participants into groups defined as having 0, 1, 2, or 3 and more comorbidities; the following common NCDs were considered: myocardial infarction, ischemic heart disease, hypertension, diabetes mellitus, hypo/hyperthyroidism, stroke, epilepsy, chronic headache, chronic kidney disease, arthritis, osteoporosis, chronic obstructive pulmonary diseases, fatty liver, cancers, asthma, gastrointestinal conditions, peptic ulcer, Crohn’s disease, ulcerative colitis, multiple sclerosis, chronic depression and psychological disorders). ‘Multi-risk factors’ was defined as having two or more of the following risk factors among obesity, hypercholesterolemia, hypertriglyceridemia, low physical activity, opium use, tobacco use, alcohol use, hookah use, high fasting blood sugar, high systolic blood pressure and high diastolic blood pressure. Logistic regression models were used to calculate crude and adjusted odds ratios (OR) and 95% confidence intervals (95% CI) for the association of socio-demographic and biochemical variables, as well as the multi-risk factors variable (independent variables) with the multimorbidity variable (either ≥2, or using the 4 categories) as the outcome. Statistical tests were two-sided, and P values less than 0.05 were considered statistically significant. Data analysis was performed using Stata software (Stata Corp. 2020. Statistical Software: Release 16. College Station, TX: Stata Corp LP).

## Results

Of the 10075 participants in the SCS, 5321 (52.8%) were female, and 3041 (30.2%) were living in rural areas. The mean age of participants at enrollment was 49.6 years. The proportion of married participants was high (93.8%). While 22.8% of the participants had a bachelor's degree or higher level of education, 32.7% were illiterate. The main socio-demographic characteristics of participants at baseline are shown in Table 2.

**Table 2:** Socio-demographic characteristics of participants at baseline of the Shahrekord PERSIAN Cohort Study, by sex and residence area

Variable	Total, N (%)	Sex		***P***	Place of residence		***P***
		Male (%)	Female (%)		Urban area (%)	Rural area (%)	
Age group (years)							
35-49	5299 (52.6)	2398 (50.4)	2901 (54.5)		3770 (53.6)	1529 (50.3)	
50-59 60-70	2996 (29.7) 1780 (17.7)	1405 (29.6) 951 (20.0)	1591 (29.9) 829 (15.6)	0.001	2091 (29.7) 1173 (16.7)	905 (29.8) 607 (20)	0.001
Sex	10075 (100)	4754 (47.2)	5321 (52.8)	-	7034 (69.8)	3041 (30.2)	-
Ethnicity							
Bakhtiari	4869 (48.3)	2236 (46.9)	2643 (49.6)		1934 (27.4)	2945 (96.8)	
Fars Turk Other	4102 (40.7) 682 (6.8) 422 (4.2)	2021 (42.4) 349 (7.3) 160 (4/3)	2088 (39.2) 336 (6.3) 262 (4.9)	0.001	4103 (58.2) 680 (9.6) 336 (4.8)	6 (0.2) 5 (0.2) 86 (2.8)	0.001
Educational attainment							
Illiterate (<1) year	3291 (32.7)	1001 (21.6)	2290 (43.7)		1335 (19.4)	1956 (65.6)	
Primary school (1-5)	1616 (16.0)	761 (16.4)	855 (16.3)		1039 (15.8)	577 (19.4)	0.001
Secondary school (6-8) High school (9-12) Bachelor’s degree (13-16) Masters/PhD degree (≥16)	995 (9.9) 1670 (16.6) 1878 (18.6) 423 (4.2)	560 (12) 924 (19.9) 1072 (23.1) 314 (6.8)	435 (8.2) 749 (14.2) 806 (15.4) 109 (2.1)	0.001	767 (11.1) 1519 (22) 1821 (26.4) 412 (6.0)	228 (7.6) 151 (5) 57 (1.9) 11 (0.4)	
Marital status							
Single	169 (1.7)	72 (1.5)	98 (1.8)		125 (1.8)	45 (1.5)	
Married Widow Divorce	9454 (93.8) 377 (3.7) 75 (0.7)	4647 (97.7) 11 (0.2) 25 (0.5)	4807 (90.3) 366 (6.9) 50 (0.9)	0.001	6672(94.9) 183(2.6) 55(0.8)	2782 (91.5) 194 (6.4) 20 (0.7)	0.001
Employment							
Employed Unemployed	4720 (46.8) 5128 (50.9)	3661 (77) 955 (20.1)	1059 (19.9) 4173 (78.4)	< 0.001	3525 (50.1) 3361 (47.8)	1195 (39.3) 1767 (58.1)	0.001
Health insurance (yes)	4820 (99.3)	2308 (99.1)	2512 (99.4)	0.275	2959 (98.9)	1861 (99.9)	< 0.001
Wealth index							
Quintiles1 (poorest)	2582 (25.7)	1013 (21.3)	1619 (30.5)		656 (9.3)	1976 (64.9)	
Quintiles 2	1772 (17.5)	743 (15.6)	979 (18.5)		1138 (16.2)	584 (19.2)	< 0.001
Quintiles 3 Quintiles 4 Quintiles 5 (richest)	1943 (19.3) 2023 (20.1) 1755 (17.4)	928 (19.5) 1004 (21.2) 1066 (22.4)	1015 (19) 1019 (19.1) 689 (12.9)	0.001	1613 (22.9) 1930 (27.5) 1697 (24.1)	330 (10.9) 93 (3.1) 58 (1.9)	

The prevalence of type 2 diabetes mellitus in the SCS was 9.8% and was higher in women (10.7%) than in men (8.8%). The prevalence of hypertension was relatively high and appeared higher in women (20.2%) than in men (13.6%). Non-alcoholic fatty liver disease and thyroid disease were frequent (14.6% and 11.4%, respectively). A history of ischemic heart disease (5.7%), stroke (0.9%), and myocardial infarction (1.3%) was more frequently reported in men than women. All NCDs appeared to be more frequent in urban than rural areas, except for gastroesophageal reflux (32.3% in rural and 29% in urban areas).

The prevalence of multimorbidity (two or more NCDs) in the SCS was 32.4% and higher in women (39.1%) than men (24.9%) and higher in urban (36.6%) than in rural areas (22.8%) (Table 3). A minority of participants reported being smokers (24.7%). The prevalence of overweight and obesity was high (43.5% and 26.9%, respectively). Approximately 57% of the participants had low physical activity levels, particularly women (59%) and participants living in an urban area (66%). The prevalence of multi-risk factors (two or more NCD risk factors) in the SCS was 61.4%, higher in men (76.3%) than women (48.8%), and higher in urban (69.2%) than in rural areas (42.7%). (Table 4). The key quantitative physiological variables in terms of mean (standard deviation) were, for BMI: 27.7 (4.6) kg/m^2^, for systolic blood pressure: 115.4 (17.3) mmHg, for fasting blood sugar: 96.7 (27.3) mg/dl, for total cholesterol: 184.1 (42.8) mg/dl and for triglyceride: 150.4 (90.5) mg/dl. Additional quantitative variables, such as biochemical and hematological measures, are shown in Table 5.

**Table 3:** Prevalence of the noncommunicable diseases (NCD) at baseline of the Shahrekord PERSIAN Cohort Study, by sex and residence area

NCD	N (%)	Sex		***P***	Residence Area		
		Male (%)	Female (%)		Urban (%)	Rural (%)	***P***
Type 2 diabetes^a^	968 (9.8)	408 (8.8)	560 (10.7)	0.002	751 (10.9)	217 (7.2)	<0.001
Hypertension^a^	1694 (17.1)	632 (13.6)	1062 (20.2)	<0.001	1275 (18.5)	419 (13.9)	<0.001
Cardiac ischemic disease	566 (5.7)	321 (6.9)	245 (4.7)	<0.001	433 (6.3)	133 (4.4)	<0.001
Myocardial infraction	127 (1.3)	100 (2.1)	27 (0.5)	<0.001	111 (1.6)	16 (0.5)	<0.001
Stroke	93 (0.9)	51 (1.1)	42 (0.8)	0.125	65 (0.9)	28 (0.9)	0.959
Renal failure	60 (0.6)	31 (0.7)	29 (0.6)	0.461	48 (0.7)	12 (0.4)	0.079
Fatty liver disease	1476 (14.9)	542 (11.7)	934 (17.8)	<0.001	1231 (17.9)	245 (8.1)	<0.001
Chronic lung disease	410 (4.1)	183 (3.9)	227 (4.3)	0.340	343 (5.0)	67 (2.2)	<0.001
Thyroid disease	1145 (11.6)	215 (4.6)	930 (17.7)	<0.001	976 (14.2)	169 (5.6)	<0.001
Kidney stone	2065 (20.8)	1189 (25.6)	876 (16.7)	<0.001	1511 (21.9)	554 (18.4)	<0.001
Rheumatic disease	470 (4.7)	139 (3.0)	331 (6.3)	<0.001	370 (5.4)	100 (3.3)	<0.001
Cancers	71 (0.7)	21 (0.05)	50 (1.0)	0.003	62 (0.9)	9 (0.3)	0.001
Gallstone	406 (4.1)	93 (2.0)	313 (6.0)	<0.001	315 (4.6)	91 (3.0)	<0.001
Multiple sclerosis	19 (0.2)	6 (0.1)	13 (0.2)	0.179	17 (0.2)	2 (0.1)	0.059
Depression	1610 (16.3)	432 (9.3)	1178 (22.4)	<0.001	1272 (18.4)	338 (11.2)	<0.001
Gastroesophageal reflux	2976 (30.0)	1229 (26.4)	1747 (33.2)	<0.001	2003 (29.0)	973 (32.3)	<0.001
Multimorbidity^b^	3273 (32.4)	1188 (24.9)	2085 (39.1)	<0.001	2578 (36.6)	695 (22.8)	<0.001
No NCDs	3983 (39.5)	2179 (45.8)	1804 (33.9)		2449 (34.8)	1534 (50.4)	
One NCDs Two NCDs ≥3 NCDs	2819 (28) 1680 (16.6) 1593 (15.9)	1387 (29.2) 703 (14.8) 485 (10.2)	1432 (26.9) 977 (18.4) 1108 (20.8)	<0.001	2007 (28.6) 1259 (17.9) 1319 (18.7)	812 (26.7) 421 (13.9) 274 (9)	<0.001

^a^ Laboratory measurements were used for definition of these NCDs

^b^ Multimorbidity; the co-existence of two or more NCDs in the cohort

**Table 4:** Prevalence of some NCD risk factors at baseline of the Shahrekord PERSIAN Cohort Study, by sex and residence area

Risk factors	N (%)	Sex		*P*	Residence Area		
		Male (%)	Female (%)		Urban (%)	Rural (%)	*P*
Tobacco use ^a^							
Frequent (%) Sometimes (%)	1275 (12.7) 271 (2.7)	1261 (27.1) 267 (5.7)	14 (0.3) 4 (0.1)	0.001	914 (13.3) 234 (3.4)	361 (12) 37 (1.2)	<0.001
Use of alcohol (yes)	1230 (12)	1213 (24.4)	17 (0.32)	0.001	1066 (15.7)	157 (5.1)	<0.001
Use of hookah (yes)	1381 (13.4)	1158 (23.3)	223 (4.2)	<0.001	1103 (15.5)	268 (8.8)	<0.001
Use of opium (yes)	1283 (12.5)	1252 (25.2)	31 (0.58)	<0.001	832 (11.7)	434 (14.2)	<0.001
Smoking (yes)	2534 (24.7)	2499 (50.3)	35 (0.6)	<0.001	1914 (27)	594 (19.5)	<0.001
Physical activity (MET)^b^							
Low (<40.4)	5744 (57.0)	2612 (54.9)	3132 (58.8)	<0.001	4639 (65.9)	1105 (36.3)	<0.001
High (≥40.4)	4331 (43.0)	2142 (45.1)	2189 (41.2)		2395 (34.1)	1936 (63.7)	
Body mass index (kg/m^2^)							
Underweight (<18.5)	123 (1.2)	80 (1.7)	43 (0.8)		61 (0.9)	62 (2.0)	
Normal (18.5-24.9) Overweight (25-29.9) Obese (≥30)	2781 (27.8) 4384 (43.8) 2710 (27.1)	1588 (33.8) 2188 (46.6) 844 (18.0)	1193 (22.5) 2196 (41.4) 1866 (35.2)	<0.001	1706 (24.5) 3238 (46.5) 1964 (28.2)	1075 (35.5) 1146 (37.8) 746 (24.6)	<0.001
Urine pH higher than 8.0	131 (1.3)	42 (0.88)	89 (1.6)	<0.001	126 (1.8)	5 (0.16)	<0.001
Hypercholesterolemia^c^ (≥240 (mg/dl))	879 (8.9)	329 (7.1)	550 (10.5)	<0.001	710 (10.3)	169 (5.8)	<0.001
Hypertriglyceridemia^d^ (≥150 (mg/dl))	3876 (39.4)	2147 (46.5)	1729 (33.1)	<0.001	3225 (46.7)	651 (22.2)	<0.001
High LDL^e^ (≥160 (mg/dl))	1915 (19.6)	743 (16.3)	1172 (22.6)	<0.001	1384 (20.2)	531 (18.2)	0.018
Multi-risk factors ^f^	5954 (61.4)	3426 (75.3)	2528 (48.8)	<0.001	4714 (69.2)	1260 (42.7)	<0.001
No risk factor	1326 (13.6)	379 (8.4)	947 (18.4)		567 (8.3)	759 (26)	
One risk factor	2439 (25)	739 (16.3)	1700 (32.8)		1525 (22.5)	914 (31.2)	
Two risk factors Three risk factors ≥ 4 risk factors	2379 (24.8) 1790 (18.4) 1785 (18.2)	1014 (22.3) 1013 (22.2) 1399 (30.8)	1365 (26.3) 777 (15) 386 (7.5)	<0.001	1758 (25.9) 1455 (21.3) 1501 (22)	621 (21) 355 (12) 284 (9.7)	<0.001

^a^ Non-cigarette tobacco (Naas, chopogh, pipe, Waterpipe)

^b^ MET, metabolic equivalent rates

^c^ high fasting plasma cholesterol

^d^ high fasting plasma triglyceride

^e^ LDL, low-density lipoproteins

^f^ Multi-risk factors; the number of simultaneous two or more risk factors of obesity/hypercholesterolemia/ hypertriglyceridemia/ low physical activity/ opium use/ tobacco use/ smoking/alcohol use/ hookah use/ high fasting blood sugar/ high systolic and diastolic blood pressure

**Table 5:** Distribution of the key quantitative variables measured at baseline of the Shahrekord PERSIAN Cohort Study

Variable	Total	Sex		***P***	Residence Area		***P***
		Male	Female		Urban	Rural	
Weight (kg)	73.4±13.4	77.3±13.4	70.0±12.4	<0.001	75.5±13.0	68.7±13.2	<0.001
Height (cm)	163.2±9.6	170.7±6.7	156.5±6.1	<0.001	164.6±9.5	159.9±9.0	<0.001
Body mass index (kg/m2)	27.6±4.6	26.5±4.1	28.6±4.9	<0.001	27.9±4.5	26.9±4.9	<0.001
Wrist circumference (cm)	17.4±1.4	18.0±1.2	16.8±1.3	<0.001	17.3±1.4	17.5±1.4	<0.001
Waist circumference (cm)	94.8±11.4	93.9±10.6	95.7±12.0	<0.001	95.1±10.8	94.2±12.6	0.001
Hip circumference (cm)	101.1±7.9	99.7±6.9	102.3±8.6	<0.001	101.9±7.7	99.3±8.2	<0.001
Education years (year)	8±6	9.6±5	6.5±5	<0.001	10±5	3.3±4	<0.001
Teeth number (n)	18.2±9.7	17.7±10.0	18.7±9.3	<0.001	19.4±9.9	15.6±8.6	<0.001
Decayed teeth (n)	1.2±2.1	1.3±2.4	1.1±1.8	<0.001	1.3±2.3	1.0±1.6	<0.001
Missing teeth (n)	13.5±9.8	14.0±10.2	13.0±9.5	<0.001	12.4±10.0	16.0±8.8	<0.001
Filled teeth (n)	3.4±4.4	2.9±4.1	3.8±4.7	<0.001	4.5±4.7	0.8±2.0	<0.001
Systolic blood pressure (mmHg)	115.4±17.3	117.9±16.6	113.2±17.7	<0.001	117.0±16.8	111.7±17.8	<0.001
Diastolic blood pressure (mmHg)	75.5±10.7	77.4±10.6	73.9±10.4	<0.001	76.3±10.8	73.8±10.1	<0.001
Pulse rate (per minute)	70.4±8	69.4±8	71.3±8	<0.001	70.9±7	69.3±9	<0.001
Fasting blood sugar (mg/dl)	96.7±27.3	97.4±26.3	96.1±28.1	0.019	102.0±27.5	84.1±22.4	<0.001
Hemoglobin (gr/dl)	14.5±1.6	15.6±1.3	13.6±1.3	<0.001	14.6±1.6	14.4±1.6	<0.001
Hematocrit (gr/dl)	42.0±4.4	44.8±3.5	39.6±3.5	<0.001	42.3±4.3	41.5±4.4	<0.001
Urine pH (value)	5.17±0.5	5.14±0.5	5.19±0.6	<0.001	5.2±0.6	5.04±0.0	<0.001
GGT (IU/L)	27.0±24.6	32.1±29.0	22.5±19.0	<0.001	28.4±26.5	23.8±19.3	<0.001
AST (IU/L)	19.6±8.8	21.2±9.7	18.3±7.5	<0.001	19.6±9.0	19.6±8.1	0.981
ALT (IU/L)	22.0±14.4	26.3±16.5	18.3±11.1	<0.001	23.3±15.2	19.1±12.0	<0.001
ALP (IU/L)	205.0±65.3	207.6±66.7	202.7±63.9	<0.001	206.8±66.6	201.0±61.9	<0.001
RBC (million/mm3)	4.8±0.5	5.0±0.5	4.5±0.5	<0.001	4.8±0.5	4.7±0.5	<0.001
WBC (thousand/mm3)	6.0±1.5	6.2±1.6	5.9±1.4	<0.001	6.0±1.5	6.0±1.5	0.195
Cholesterol (mg/dl)	184±42	180±42	187±42	<0.001	189±41	171±42	<0.001
Triglyceride (mg/dl)	150±90	163±32	139±81	<0.001	163±95	118±66	<0.001
HDL (mg/dl)	50±1	47±10	53±12	<0.001	52±11	46±10	<0.001
LDL (mg/dl)	103±33	100±31	106±34	<0.001	104±32	101±36	<0.001

*Abbreviations*: *GGT* Gamma-glutamyl transferase, *AST* aspartate aminotransferase, *ALT* alanine aminotransferase, *ALP* alkaline phosphatase, *RBC* Red blood cell, *WBC* white blood cells, *HDL* high-density lipoproteins, *LDL* low-density lipoproteins

The logistic regression analysis showed that the prevalence of multimorbidity was higher in participants living in an urban area compared with living in a rural area (OR=1.79, 95% CI: 1.53-2.09), in those aged ≥60 years old compared with 35-49 years (OR=2.38, 95% CI: 1.56-3.40) and in females compared with males (OR=2.27, 95%CI: 1.96-2.62). Physical activity was inversely associated with multimorbidity prevalence (OR=0.97, 95% CI, 0.97-0.98). We also found a significant relationship between household wealth and the presence of multimorbidity. Multimorbidity prevalence was substantially higher in participants who cumulated two or more NCD risk factors. Alcohol consumption was not associated with multimorbidity prevalence after adjustment for other exposures (P=0.78). Opium use was not associated with NCD prevalence after adjustment for other exposures (OR=1.10, 95%CI: 0.93-1.29, P=0.23). All crude and adjusted ORs for associations with multimorbidity are shown in Table 6.

**Table 6:** Crude and adjusted logistic regression models of the risk of multimorbidity in the Shahrekord PERSIAN Cohort Study

Variables	Crude - OR	CI 95 %	***P***-value	Adjusted OR	CI 95 %	***P***-value
**Sociodemographic**						
Age group (years)						
35-49 50-59 60-70	Reference 2.69 3.64	1 2.44-2.98 3.25-4.09	- 0.001 0.001	- 1.88 2.38	- 1.42-2.50 1.56-3.40	- 0.001 0.001
Sex						
Female	1.85	1.69-2.01	0.001	2.27	1.96-2.62	0.001
Residence area						
Living in rural Living in Urban	Reference 1.98	1 1.79-2.19	- 0.001	- 1.79	- 1.53-2.09	- 0.001
Ethnicity						
Fars Bakhtiari Turk Other	Reference 0.69 0.97 1.18	1 0.63-0.76 0.82-1.16 0.96-1.45	- 0.001 0.805 0.109	- 1.02 1.06 1.41	- 0.90-1.15 0.88-1.28 1.12-1.78	- 0.735 0.506 0.030
Education years (year)	0.94	0.94-0.95	0.001	0.99	0.97-1.23	0.078
Unemployment	2.10	1.12-2.29	0.001	1.16	1.02-1.32	0.017
Marital status						
Single Married Widow Divorce	Reference 1.86 3.72 1.56	1 1.26-2.75 2.40-5.77 0.82-2.97	- 0.001 0.001 0.175	- 1.27 1.24 1.08	- 0.84-1.92 0.77-1.99 0.53-2.17	- 0.244 0.366 0.821
Wealth index						
Quintile 1(poorest) Quintile 2 Quintile 3 Quintile 4 Quintile 5 (richest)	Reference 1.39 1.20 1.25 1.18	1 1.22-1.59 1.05-1.37 1.10-1.42 1.03-1.35	- 0.001 0.005 0.001 0.013	- 1.32 1.30 1.44 1.50	- 1.10-1.59 1.08-1.56 1.19-1.74 1.21-1.85	- 0.002 0.005 0.001 0.001
**Behavior/ lifestyle factors**						
Tobacco use (yes) ^a^	0.74	0.67-0.82	0.001	1.08	0.95-1.24	0.194
Use of alcohol (yes)	0.75	0.67-0.84	0.001	1.02	0.87-1.19	0.782
Use of hookah (yes) ^*^	0.69	0.53-0.90	0.007	**	-	-
Use of opium (yes) ^*^	0.86	0.76-0.96	0.01	1.10	0.93-1.29	0.233
Smoking (yes) ^*^	1.40	1.20-1.60	0.001	0.96	0.82-1.13	0.689
Physical activity (MET)	0.96	0.96-0.97	0.001	0.97	0.97-0.98	0.001
Obesity (yes)^*^	2.11	1.93-2.32	0.001	1.45	1.30-1.61	0.001
**Anthropometric/ vital values**						
Body mass index (kg/m^2^)	1.10	1.09-1.11	0.001	**	-	-
Systolic blood pressure (mmHg)	1.04	1.03-1.04	0.001	1.00	1.00-1.01	0.002
Diastolic blood pressure (mmHg)	1.04	1.04-1.05	0.001	1.01	1.00-1.01	0.011
Fasting blood sugar (mg/dl)	1.01	1.01-1.02	0.001	1.01	1.00-1.01	0.001
Hypercholesterolemia (yes) ^*^ (≥240 (mg/dl)	1.32	1.14-1.52	0.001	**	-	-
Hypertriglyceridemia^c^ (yes) ^*^ (≥150 (mg/dl))	1.48	1.36-1.61	0.001	1.13	1.02-1.25	0.015
High LDL^d^ (≥160 (mg/dl))	0.99	0.99-1.0	0.001	**	-	-
Alkaline urine pH (>8 )	1.22	1.13-1.31	0.001	1.10	1.02-1.19	0.014
**Multi-risk factors**						
None	Reference	1	-	-	-	-
One Two ≥3	1.34 1.96 2.09	1.14-1.57 1.68-2.30 1.80-2.42	0.001 0.001 0.001	1.14 1.42 1.46	0.92-1.42 1.12-1.80 1.11-1.94	0.212 0.004 0.007

^a^Non-cigarette tobacco (Naas, chopogh, pipe, Waterpipe)

^*^the reference group was the absence of the risk factor

^**^ to reduce collinearity, use of hookah, body mass index, hypercholesterolemia and high LDL, not in the multiple regression models

## Discussion

Despite the significant impact of NCDs on the health and economy of Iran, these have not yet received sufficient attention from public health institutions and the general population in southwest Iran. One possible reason for this is the lack of epidemiological data on NCDs' prevalence, multimorbidity and risk factors in developing countries, especially Iran. The study's main strength is the collection of a comprehensive set of variables and biological measures in a population including, for the first time, Bakhtiari people (about half of the cohort) and living in the highest-altitude region of Iran. The inclusion of different ethnic groups (Bakhtiari, Fars, and Turk) will allow examining the prevalence and incidence of NCDs across genetic, social, and cultural characteristics of the participants and their interaction with lifestyle and environmental exposures. The SCS biobank provides the infrastructure necessary for the long-term preservation of biological samples (whole blood, blood plasma, hair, nail, and urine) collected in all participants. The SCS is also an opportunity for increased cooperation between academic, healthcare and political systems and will permit better health-related programs. It also creates ample opportunities for the education and training of students and researchers at the Shahrekord University of Medical Sciences and collaboration with other medical research initiatives such as clinical trials and national and international health research consortia. Our previous profile on respiratory diseases [31] and the results of this study will provide meaningful information for researchers to identify relevant research questions and for health care system planners to identify priority targets for improvements.

Participants in the SCS were aged 35-70 years at baseline; therefore, it will not be possible to study NCDs in children and young adults, which constitute an essential fraction of the Iranian population. However, after obtaining information in the follow-up process, longitudinal studies and studies of older adults will be possible. Self-reported data on smoking, hookah smoking, alcohol consumption, and intake of drugs may be prone to under-reporting because of the socio-cultural characteristics of the Iranian population. The interview-based nature of some collected variables may result in underestimating a few behavioral risk factors due to socially undesirable responses. Behavioral risk factors assessed depended on the participants' ability to recall their health behaviors which might have led to recall bias. However, interviews were conducted by trained investigators, using standardized questionnaires and techniques that facilitate recall of health risk behaviors, which helped minimize these biases.

Comparing our results with other cohort studies in Iran, it is clear that the pattern of NCD prevalence, multimorbidity and risk factors differs from those observed in other regions [11, 14]. For example, the Ravansar cohort study [14] reported a higher prevalence of NCDs in women than in men, except for the high majority of kidney stones observed in men. In our study, cardiovascular disease, myocardial infarction, stroke, and kidney stones were more common in men than women. The prevalence of diseases such as chronic lung disease, non-alcoholic fatty liver, thyroid disease, hypertension and diabetes was higher in our study than in the Ravansar study [14]. Our population sample differs from that included in the AZAR cohort [15] conducted in Tabriz in northwestern Iran (another PERSIAN cohort center) in terms of NCD prevalence. The prevalence of hypertension in the AZAR cohort was 25%, substantially higher than in the SCS (17%). The prevalence of diabetes (14%) in the AZAR cohort study was 4% higher than ours. On the contrary, the prevalence of chronic lung disease and thyroid diseases appear to be higher in our cohort than in the AZAR cohort. Several differences can also be highlighted with other PERSIAN cohort centers, such as the Rafsanjan cohort [32] and the Hoveyzeh Cohort Study (HCS), a prospective population-based study on non-communicable diseases in an Arab community of Southwest Iran [33]. For example, the prevalence of diabetes, hypertension, obesity, cardiovascular disease, and stroke in the Arab ethnic group of the Hoveyzeh cohort is higher than in our study [33]. The prevalence of NCD multimorbidity in this study was 32.4%, close to a third of the participants. This was somewhat lower than in the Kurdish population and more than in the Golestan cohort study in Iran, which reported a prevalence of 36.6% and 19.4%, respectively [34, 35]. Future research using the SCS data will investigate reasons for such discrepancies in NCD prevalence and risk factors across Iran, considering both environmental, racial, biological and genetic factors involved in NCDs, which may vary and require different interventions across regions. Although the questionnaires and tools we used were the same, or similar, in our study and other PERSIAN cohorts, additional research is required before formal conclusions are drawn about differences between the SCS and other Iranian regions.

## Conclusion

The Shahrekord Cohort Study (SCS), part of the PERSIAN Cohort, is a unique, comprehensive, and large-scale study conducted for the first time in Chaharmahal and Bakhtiari province, characterized by its large Bakhtiari ethnic group (about half of the participants) and its high altitude. The study collected data on a large number of exposures such as demographics, lifestyle, dietary habits, employment, social integration, quality of life (e.g., sleep patterns, stress), family medical history and use of medication, and the number of anthropometric and physiological measures (e.g., respiratory capacity tests, electrocardiograms). A rich biobank was created by collecting, in all cohort participants, samples from whole blood, serum, plasma, buffy coat, hair, nail, and urine. Diseases were ascertained from clinical examinations, biochemical variables, interviews, and linkage with medical records registered in the integrated health information system. The SCS provides a platform for epidemiological studies that will be useful for better prevention and management of NCDs in the southwest of Iran. Preliminary analysis of the baseline data highlights the high prevalence of NCD multimorbidity (one-third of the population) and risk factors for NCDs in the Chaharmahal and Bakhtiari province. Considering the high prevalence of overweight, hypertriglyceridemia, fatty liver disease, hypertension, thyroid disease, and cardiovascular disease, the SCS will be an essential resource for better identifying NCD risk factors in this region and designing relevant public health interventions. We expect that our findings will help improve health in the Chaharmahal and Bakhtiari province, by providing public health decision-makers with accurate information to develop policies to improve the diagnosis, management and control of NCDs.

## Data Availability

The SCS data are not open-access, but external investigators may use the data for collaborative projects. Information relative to data access and collaboration can be obtained from the corresponding author Dr. Ali Ahmadi, or at info@persiancohort.com. Suggested projects are first discussed by SCS principal investigators, and the final decision on data sharing for national and international collaborative projects is made by the SCS scientific committee. Further details about the cohort and information relative to data access, collaborative research, and publications can be found at http://persiancohort.com/cohortsites/shahrekord or on the SCS website https://cohort.skums.ac.ir.

## Abbreviations

NCDs: Non-communicable diseases
WHO: World Health Organization
PERSIAN: Prospective Epidemiological Research Studies in IrAN
CH & B: Chaharmahal and Bakhtiari
M.S.: Multiple sclerosis
DMFT: Decayed, Missing and Filled Teeth
HIS: Hospital Information System
MOHME: Ministry of Health and Medical Education
MET: Metabolic equivalent rates
LDL: Low-density lipoproteins
FBS: Fasting blood sugar
GGT: Gamma-glutamyl transferase
AST: Aspartate aminotransferase
ALT: Alanine aminotransferase
ALP: Alkaline phosphatase
RBC: Red blood cell
WBC: White blood cells
